# SARS-CoV-2 Transmission between Mink (*Neovison vison*) and Humans, Denmark

**DOI:** 10.3201/eid2702.203794

**Published:** 2021-02

**Authors:** Anne Sofie Hammer, Michelle Lauge Quaade, Thomas Bruun Rasmussen, Jannik Fonager, Morten Rasmussen, Karin Mundbjerg, Louise Lohse, Bertel Strandbygaard, Charlotte Sværke Jørgensen, Alonzo Alfaro-Núñez, Maiken Worsøe Rosenstierne, Anette Boklund, Tariq Halasa, Anders Fomsgaard, Graham J. Belsham, Anette Bøtner

**Affiliations:** University of Copenhagen, Copenhagen, Denmark (A.S. Hammer, M.L. Quaade, K. Mundbjerg, A. Boklund, T. Halasa, G.J. Belsham, A. Bøtner);; Statens Serum Institut, Copenhagen (T.B. Rasmussen, J. Fonager, M. Rasmussen, L. Lohse, B. Strandbygaard, C.S. Jørgensen, A. Alfaro-Núñez, M.W. Rosenstierne, A. Fomsgaard, A. Bøtner)

**Keywords:** Coronavirus, full-genome sequence, virus adaptation, virus transmission, mink, Denmark, 2019 novel coronavirus disease, SARS-CoV-2, severe acute respiratory syndrome coronavirus 2, zoonoses, coronavirus disease, COVID-19, viruses, respiratory infections

## Abstract

Severe acute respiratory syndrome coronavirus 2 has caused a pandemic in humans. Farmed mink (*Neovison vison*) are also susceptible. In Denmark, this virus has spread rapidly among farmed mink, resulting in some respiratory disease. Full-length virus genome sequencing revealed novel virus variants in mink. These variants subsequently appeared within the local human community.

Severe acute respiratory syndrome coronavirus 2 (SARS-CoV-2) has caused the ongoing coronavirus disease (COVID-19) pandemic (*1*). Ferrets, cats, dogs, Syrian hamsters, and nonhuman primates can be infected with the virus and, in some cases, transmit it (*2*); however, other species, such as pigs and chickens, appear resistant (*3**,**4*). Thus, the virus has a restricted host range. Infection with SARS-CoV-2 has occurred in farmed mink in the Netherlands (*5*).

In Denmark, there are »1,200 mink farms (*6*). Because of contacts between persons with COVID-19 and mink farms, investigation of SARS-CoV-2 infection within mink in Denmark was undertaken. We documented 3 premises in the Northern Jutland region of Denmark with SARS-CoV-2–infected mink and analyzed virus transmission in mink and the local human community.

## The Study

We collected blood and throat, nasal, and fecal swab samples from mink adults and kits (Table 1); we also sampled feed and air. We assayed viral RNA by quantitative reverse transcription PCR (qRT-PCR) (*7*). We performed SARS-CoV-2 Ab ELISA (Beijing Wantai Biological Pharmacy Enterprise, http://www.ystwt.cn) as described (R. Lassaunière et al., unpub. data, https://doi.org/10.1101/2020.04.09.20056325). SARS-CoV-2–positive RNA samples were sequenced and sequences aligned using Mafft (https://mafft.cbrc.jp/alignment/server/index.html). Phylogenetic analysis was performed in MEGA 10.1.7 (*8*) using the maximum-likelihood general time reversible plus invariant sites plus gamma (2 categories) method (*9*).

**Table 1:** Summary of laboratory analyses of mink samples from 3 mink farms tested for severe acute respiratory syndrome coronavirus 2 in Denmark, June–July 2020*

Sample origin	Test and specimen type, no. positive/no. tested (%)						Date of sample collection	Location
	ELISA		qRT-PCR					
	Serum		Throat swabs		Nasal swabs	Fecal swabs		
Live adult mink	29/30 (97)		NA	NA		5/30 (17)	2020 Jun 14	Farm 1
Dead adult mink	NA		NA	4/4 (100)		3/4 (75)	2020 Jun 14	Farm1
Live mink kits	30/30 (100)		3/30 (10)	3/30 (10)		1/30 (3)	2020 Jun 17	Farm 1
Live adult mink	30/30 (100)		3/23 (13)	NA		0/23 (0)	2020 Jun 17	Farm 1
Retested adult mink	4/4 (100)		2/4 (50)	2/4 (50)		1/4 (25)	2020 Jun 17	Farm 1
Live adult mink	1/30 (3)		NA	NA		0/8 (0)	2020 Jun 18	Farm 2
Dead adult mink	NA		1/8 (13)	NA		NA	2020 Jun 18	Farm 2
Live mink kits	1/50 (2)		40/50 (80)	39/50 (78)		NA	2020 Jun 22	Farm 2
Live adult mink	3/50 (6)		46/50 (92)	NA		NA	2020 Jun 22	Farm 2
Dead adult mink	1/3 (33)		2/3 (66)	2/3 (66)		NA	2020 Jun 22	Farm 2
Dead adult mink	NA		3/3 (100)	3/3 (100)		NA	2020 Jun 30	Farm 2
Live adult mink (retest)	36/37 (97)		35/37 (95)	37/37(100)		NA	2020 Jun 30	Farm 2
Live adult mink	20/30 (67)		6/6†(100)	NA		NA	2020 Jun 30	Farm 3
Dead adult mink	NA		5/5 (100)	NA		NA	2020 Jun 30	Farm 3
Live mink kits	24/30 (80)		30/30 (100)	27/30 (90)		NA	2020 Jul 2	Farm 3
Live adult mink	23/30 (77)		30/30 (100)	26/30 (87)		NA	2020 Jul 2	Farm 3

*NA, not applicable; qRT-PCR, quantitative reverse transcription PCR. †Samples from 30 mink were assayed in 6 pools of 5 swabs each.

We selected mink farms for investigation because of COVID-19 in persons linked to them. During initial visits, we sampled 30 apparently healthy adult mink; we tested adults and kits in follow-up visits. We analyzed serum samples for SARS-CoV-2 antibodies and assayed swab samples for SARS-CoV-2 RNA (Table 1; Appendix). At initial sampling, seroprevalence was high on farm 1 (>95%) and farm 3 (66%) but, in contrast, only 3% on farm 2. However, after the infection spread widely on farm 2, indicated by the increased prevalence of viral RNA (Table 1), a large increase in seroprevalence occurred, to >95%.

Air samples from farm 1 tested negative. However, on farms 2 and 3, multiple samples collected from exhaled air from mink or within 1 m of the cages scored positive, albeit with fairly high (>31) C_t_ values. None of the air samples collected outside the houses were positive. Feed samples collected at each farm tested negative.

We also sequenced SARS-CoV-2 RNA from samples from each mink farm. The viruses found on farms 1–3 were very similar (Table 2). These sequences and those from humans (H1–H9) linked to the infected farms grouped within the European 20B clade of the global SARS-CoV-2 tree (*10**,**11*) (Figure; Appendix Table 1). We deposited the SARS-CoV-2 genome sequences of virus from farm 1 (SARS-CoV-2/mink/DK/AD3_Farm1/2020) in GenBank (accession nos. MT919525–36). The sequences closely matched those of a human case, diagnosed in mid-May, with a direct epidemiologic link to farm 1. This index sequence (only 91% complete) matched the mink viruses at nt 15656 (rare globally) but had A at nt 22920 (Table 2). The nt 25936 in the index case could not be determined. The local phylogeny (Appendix Figure) showed that mink sequences from farm 1 fell into 3 subclusters (defined by the nucleotide changes at positions 5421 and 22920), but sequences from linked humans (H1–H9) and mink in farms 2 and 3 were within subcluster 2 (Appendix Figure).

**Table 2:** Location of nt differences identified in genome sequences of selected severe acute respiratory syndrome coronavirus 2 samples from mink and humans in Denmark, June–July 2020, compared with Wuhan and clade 20B reference sequences*

Virus sample	Genomic location and nt position																
	5´ UTR		ORF1a				ORF1b			Spike			ORF3a		Nucleoprotein		
	241		3037	5421	9534		14408	15656		22920	23403		25936		28881	28882	28883
NC045512 (Wuhan)	C		C	A	C		C	C		A	A		C		G	G	G
Humans in Jutland (to 2020 Jun 10)†	T		T	A	C		T	C		A	G		C		G	G	G
EPI_ISL_455326 20B	T		T	A	C		T	C		A	G		C		A	A	C
Index case	T		T	A	C		T	T		A	G		ND		A	A	C
Mink_AD4_ Farm1	T		T	G	C		T	T		T	G		T		A	A	C
Mink_AL3_ Farm1	T		T	A	C		T	T		A	G		T		A	A	C
Mink_KL14_ Farm1	T		T	A	C		T	T		A	G		T		A	A	C
Mink_KL11_ Farm1	T		T	A	C		T	T		A	G		T		A	A	C
Mink_AD3_ Farm1	T		T	G	C		T	T		T	G		T		A	A	C
Mink_AD6_ Farm1	T		T	A	C		T	T		T	G		T		A	A	C
Mink_AL64_ Farm1	T		T	A	C		T	T		A	G		T		A	A	C
Mink_AL25_ Farm1	T		T	A	C		T	T		T	G		T		A	A	C
Mink_AD38_ Farm2	T		T	A	C		T	T		T	G		T		A	A	C
Mink_M1-M47_Farm2‡	T		T	A	C		T	T		T	G		T		A	A	C
Mink_AD37_ Farm3	T		T	A	C		T	T		T	G		T		A	A	C
Mink_AD40_ Farm3	T		T	A	C		T	T		T	G		T		A	A	C
Mink_AL35_ Farm3	T		T	A	C		T	T		T	G		T		A	A	C
H1–H7 + H9	T		T	A	C		T	T		T	G		T		A	A	C
H8	T		T	A	T		T	T		T	G		T		A	A	C
In NB01 (NL)§	T		T	A	C		T	C		A	G		C		G	G	G
In NB02 (NL)§	C		C	A	C		C	C		T>A#	A		C		G	G	G
In NB03 (NL)§	T		T	A	C		T	C		A	G		T		G	G	G
In NB04 (NL)§	T		T	A	C		T	C		A	G		C		G	G	G
Humans in Jutland (to 2020 Jul 1) †	T		T	A	C		T	C>T		A>T	G		C>T		G>A	G>A	G>C
Encoded amino acid change¶	NA		NA	I1719 V	T3083 I		P314 L	T730 I		Y453F	D614 G		H182 Y		R203 K	R203 K	G204 R

*Red text indicates nt differences from the Wuhan reference strain; pink shading indicates nt changes detected in mink and in human contacts (H1–H9) that differ from the clade 20 B and index case; gray shading indicates a reference clade 20B sequence and the human index case sequence. NA, not applicable, as nt change in the noncoding region; ND, not determined; NL, the Netherlands; ORF, open reading frame. † The proportions of each nt present at each of these positions in human sequences in Jutland are shown in Appendix Table 1 (https://wwwnc.cdc.gov/EID/article/27/2/20-3794-App1.pdf). ‡nts present in farm 2 sequences obtained from throat swab specimens on June 22, 2020 (derived from 20 adult mink and 27 kits). §The mink sequences from the Netherlands also differ at other locations compared with the Wuhan sequence (*5*). ¶Encoded amino acid substitutions (with residue number in each protein) compared to Wuhan reference strain are indicated using the single letter code. #T in 5 of 6 sequences from farm NB02 (*5*).

**Figure:**
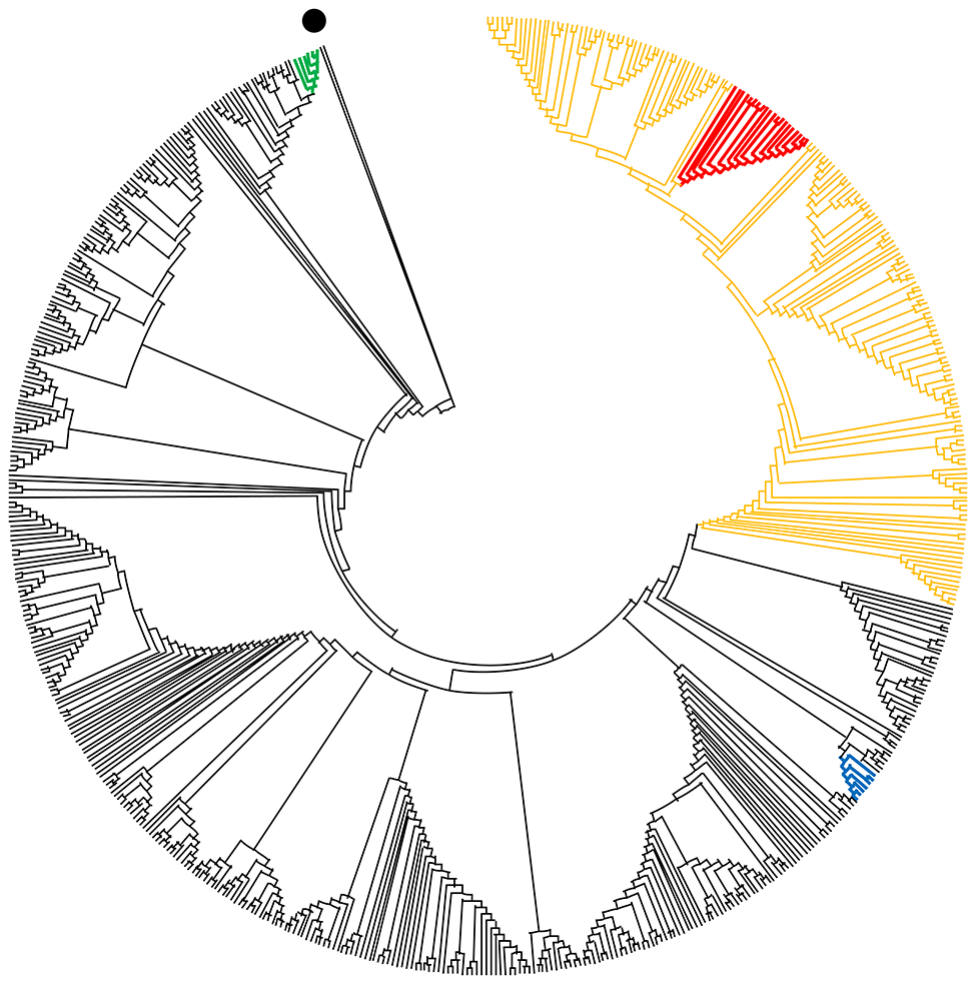
Phylogenetic tree showing relationships between genome sequences of severe acute respiratory syndrome coronavirus 2 from mink and humans at 3 mink farms in Denmark, June–July 2020 (red), and selected global full-length genome sequences. Black dot indicates Wuhan reference sequence NC_045512.2; green indicates mink farm NB02 in the Netherlands; blue indicates mink farms NB01, NB03, and NB04 in the Netherlands; orange indicates clade 20B.

We found 9 to 11 nt differences (mainly nonsynonymous) between the mink sequences in Denmark and the Wuhan-Hu-1 reference sequence (Table 2). One mutation at nt 23403 (resulting in substitution D614G in the spike protein) was present in all sequences from mink in Denmark and the Netherlands, except for NB02 from the Netherlands (Table 2) and was predominant in the human population in Jutland (Appendix Table 1) and globally (*12*). However, another mutation (nt C25936T [as cDNA] encoding H182 to Y within ORF3a) appeared in all mink sequences from Denmark (Table 2) and in human cases (H1–H9) linked to them. This change was not found in human SARS-CoV-2 sequences from Jutland before June 10, 2020 (Appendix Table 1), but reached »40% frequency during June 10–July 1, 2020 (Table 2; Appendix Table 2). This mutation has been found only rarely in other SARS-CoV-2 sequences (*11*) (Appendix Table 1) but was in mink farm NB03 from the Netherlands (SARS-CoV-2/mink/NED/NB03_index/2020; GenBank accession no. MT457400.1).

another mutation in the spike gene (A22920T, encoding Y453 to F) was present in 4 of 8 sequences from farm 1, in all sequences from farms 2 and 3, and in 5 of 6 sequences from farm NB02 in the Netherlands (*5*). This change was not in the index case or the human population anywhere before June 10 but was subsequently detected in farm-linked humans (H1–H9) and in Jutland (Table 2; Appendix Table 2). Finally, the mutation in the open reading frame 1b gene (C15656T, encoding T730 to I) was present only in mink/human sequences from Denmark (Table 2) and a sequence from New Zealand (Appendix Table 1).

## Conclusions

A high proportion of mink on farms can be infected with SARS-CoV-2 within a few days, which may provide major virus exposure to persons working with mink. The infections we describe here occurred with little clinical disease or increase in death (Appendix), making it difficult to detect the spread of infection; thus, mink farms could represent a serious, unrecognized animal reservoir for SARS-CoV-2. There is no evidence for spread of the virus outside of farm buildings, either in Denmark or in the Netherlands (*5*), except by infected persons. However, there appears to be some risk of virus transmission to persons working with infected mink as well as for their contacts and thus, indirectly, for the public.

On farm 1, the virus had probably been introduced some weeks before detection (Table 1). On farm 2, the low frequency (4%) of seropositivity and the high proportion of qRT-PCR positive animals at second sampling (Table 1) suggested that the virus had been recently introduced but was spreading. Indeed, a third sampling (8 days later) showed a much higher seroprevalence (>90%). Conceivably, the variant viruses that appeared in farm 1 and spread to farms 2 and 3 may be better adapted to mink and thus able to transmit rapidly. The infection at farm 3 was detected relatively late, with a high seroprevalence (66%) at first visit.

A likely scenario for the spread of infection in mink in Denmark is that the index human case-patient who had nt T15656 introduced it into farm 1. Initially, we observed sequence heterogeneity at nt 22920 in mink on farm 1, but subsequently, we detected only the variant form (T22920) on farms 2 and 3 and in subsequent linked human cases (H1–H9) (Table 2). Remarkably, this heterogeneity also occurred on farm NB02 in the Netherlands. This change, possibly together with the mutation at nt 25936 (Table 2), may represent virus adaptation. It is not yet established whether these changes confer advantages in mink, but the variant viruses in farm 2 spread rapidly. It seems that the variant viruses on farm 1 spread to >1 human and were then transmitted, presumably by human–human contact, to other persons and to farms 2 and 3. The change at nt 22920 results in substitution Y453F in the S-protein (Table 2). This Y-residue, within the receptor-binding motif of the S-protein, is highly conserved among SARS-related coronaviruses and is close to residue L455 that is critical for interaction with the cellular ACE2 receptor (*13*).

Appendix Additional information about SARS-CoV-2 transmission between mink and humans, Denmark.
