# Supplementary material for: SARS-CoV-2 Transmission between Mink (Neovison vison) and Humans, Denmark
Source: Emerg Infect Dis. 2021 Feb;27(2):547–51. doi: 10.3201/eid2702.203794 (PMC7853580; doi:10.3201/eid2702.203794)
Supplement: Appendix — Additional information about SARS-CoV-2 transmission between mink and humans, Denmark. [file 20-3794-Techapp-s1.pdf]

Article DOI: https://doi.org/10.3201/eid2702.203794

SARS-CoV-2 Transmission between Mink (*Neovison vison*) and Humans, Denmark

Appendix

Additional Materials and Methods

RT-qPCR

Viral RNAs were extracted from throat, nasal, and fecal swab samples and feed using a MagNaPure 96 robot with the MagNaPure 96 DNA and Viral Small volume kit (Roche, https://www.roche.com) and assayed using the E gene RT-qPCR assay (*1*). Air samples were collected using an AeroCollect electrostatic air sampler (FORCE Technology, https://forcetechnology.com). Material captured on filters, during a period of 10–15 min, by the air samplers, was eluted using nuclease-free water and tested directly without prior extraction of RNA. Briefly, eluted samples were screened using the E gene reverse transcription quantitative PCR (RT-qPCR) assay but with the Luna Universal Probe One-Step RT-qPCR Kit, 2x Luna WarmStart RT Enzyme Mix (20X) with added IGEPAL CA-630 (0.3%) (Sigma-Aldrich, https://www.sigmaaldrich.com).

Whole Genome Sequencing and Data Analysis

cDNA synthesis was performed with SuperScript IV First Strand Synthesis Kit (Thermo Fisher Scientific, https://www.thermofisher.com) according to the manufacturer’s specifications. Amplification of the viral cDNA and preparation of the Nanopore sequencing libraries was performed according to the ARTIC sequencing protocol v2 (https://artic.network/ncov-2019). Sequence libraries were loaded into a FLO-MIN 106 flow cell and sequenced on a MinION device (Oxford Nanopore Technologies, https://nanoporetech.com). High-accuracy base calling was performed using Guppy v3.4.5 (Oxford Nanopore). Reads were imported into CLC Genomics Workbench version 20.04.4 (http://resources.qiagenbioinformatics.com). Mapping of long reads against the Wuhan reference sequence (NC_045512.2) was performed using the Long Read support version 20 β 1 (https://digitalinsights.qiagen.com/plugins/long-read-support). Raw

reads were trimmed versus the ARCTIC protocol primers and end-trimmed (30 nt). Consensus sequences were generated with a minimum depth of 10 and use of quality-based vote consensus building. All positions with less than 90% site coverage were excluded from the analysis.

A subset of 567 reference sequences used in Nextstrain on June 26, 2020 were downloaded from GISAID; these were selected based on quality and matching the proportionate distribution of the major clades to the set of sequences used in Nextstrain.

The mink-derived sequences and linked human cases belonged to European Clade 20B. This is specifically defined by the mutations G28881A, G28882A, and G28883C and supported by the presence of the mutations C3037T and C14408T (Table 2 in the main article).

Farm Sampling

Sampling and Testing of Mink in Farm 1

Farm 1 was in northern Jutland. It had ≈1,800 adult females and >9,000 kits, born around the beginning of May 2020. No excess deaths had occurred but some mild respiratory distress had been observed in a few adult mink. Initially, fecal and nasal swab samples as well as blood samples were collected (Table 1 in the main article). Five of 30 live mink and all 4 deceased mink scored positive by RT-qPCR (Table 1). Furthermore, 29 of 30 animals scored positive by ELISA for anti–SARS-CoV-2 antibodies (Table 1). In contrast, 11 stored mink serum samples, collected in 2016 before the emergence of SARS-CoV-2 in 2019, scored negative in this assay.

In follow-up sampling, 4 of 30 mink kits tested scored SARS-CoV-2 RNA positive (Table 1). Serum samples from all 30 kits and 30 adults scored positive by ELISA. In retesting of 4 adult females that tested positive by RT-qPCR in the initial testing, 3 again tested positive and all 4 were again scored as seropositive (Table 1). Samples of mink feed and of air samples collected from the farm all proved negative in RT-qPCR assays.

Sampling and Analysis of Mink at Farm 2

Farm 2, located ≈12 km from farm 1, had ≈700 adult female mink and 3,500 kits. One person on this farm, who had contact with the mink, tested positive for SARS-CoV-2 and was linked to infected persons connected to farm 1. Initial sampling from the mink occurred on June 18, 2020, when no clinical signs or excess deaths had been observed. Serum samples from 30 adult mink were assayed by ELISA and one scored positive (Table 1). In addition, samples from

8 live and 8 dead mink were tested by RT-qPCR; 1 throat swab tested strongly positive (C_t = 20.4) but none of the rectal swabs did (Table 1). During a follow-up visit on June 22, 40 of the 50 swab samples from mink kits were scored positive by RT-qPCR (Table 1). However, only 1 of the serum samples from these 50 kits tested positive by ELISA. In the adult animals, 46 of 50 throat swab samples were RT-qPCR positive but only 3 serum samples from these animals scored positive by ELISA (Table 1). One of 3 sick mink, with respiratory symptoms, scored strongly positive from testing the throat swab sample (C_t = 17.6) and was also seropositive. In addition, an air sample, collected directly from the exhaled air of this mink, scored positive (C_t = 31.4).

In the following days, several mink displayed respiratory disease symptoms. On June 30, adult females sampled previously were resampled and some had clear respiratory symptoms and distress. In a period of 8 days, the seroprevalence among the adult mink increased from 6% to >95% and 35 of 37 throat swab samples were again positive by RT-qPCR (Table 1). During June 22–30, 4 of the 50 animals had died. Three of these had high levels of SARS-CoV-2 RNA (C_t <25) in throat swabs; the fourth was unavailable. Three separate mink feed samples were scored negative.

Sampling and Analysis of Mink at Farm 3

Farm 3, with ≈1,000 adult females and almost 4,500 kits is located <1 km from farm 1, and 1 person in contact with the mink had tested positive for SARS-CoV-2. Some clinical signs of respiratory disease had been apparent and 20–30 mink had died in the 2 weeks before initial sampling on June 29, 2020. By ELISA, 20 of the 30 serum samples tested positive (Table 1), suggesting that the infection had been introduced ≥1–2 weeks before this sampling. Throat swabs from 30 live mink were tested in pools; all 6 pools were positive by RT-PCR, as were throat swabs from 5 dead mink. On July 2, during follow-up, 23 of the 30 adult mink were seropositive, as were 24 of the 30 mink kits. All the throat swabs and most of the nasal swabs scored positive by RT-qPCR (Table 1). Air samples collected from near the mink (<1 m from cages) were also positive, but air samples from outside the open buildings (2–3 m from cages) were negative. The mink on farms 1, 2, and 3 have all been culled.

Infection of mink by SARS-CoV-2 has now been detected in Spain (https://promedmail.org/promed-post/?id = 20200717.7584560) and in the United States (http://www.promedmail.org/post/7692815).

Reference

<jrn>1. Corman VM, Landt O, Kaiser M, Molenkamp R, Meijer A, Chu DK, et al. Detection of 2019 novel coronavirus (2019-nCoV) by real-time RT-PCR. Euro Surveill. 2020;25:2000045. https://doi.org/10.2807/1560-7917.ES.2020.25.3.2000045</jrn>

Appendix Table 1. Sequence differences between selected samples from mink and humans in Denmark along with reference sequences for defined clades (see also Table 2 in the main article)*

	Genomic position (nt)											
	5′UTR	ORF1a			ORF1b		S		ORF3a	N		
Sequence	241	3037	5421	9534	14408	15656	22920	23403	25936	28881	28882	28883
New Zealand /EPI_ISL_456190	C	C	A	C	C	T	A	A	C	G	G	G
Egypt /MT611448.1	T	T	A	C	T	C	A	G	T	G	G	G
Saudi Arabia /EPI_ISL_437692	T	T	G	C	T	C	A	G	C	G	G	G
Sweden /EPI_ISL_469065	T	T	A	C	T	C	A	G	T	A	A	C
Mali /EPI_ISL_487452	C	C	A	C	C	C	A	A	T	G	G	G
EPI_ISL_415504 19A	C	C	A	C	C	C	A	A	C	G	G	G
EPI_ISL_435126 19A	C	C	A	C	C	C	A	A	C	G	G	G
EPI_ISL_408670 19A	C	C	A	C	C	C	A	A	C	G	G	G
EPI_ISL_416885 19B	C	C	A	C	C	C	A	A	C	G	G	G
EPI_ISL_408668 19B	C	C	A	C	C	C	A	A	C	G	G	G
EPI_ISL_407193 19B	C	C	A	C	C	C	A	A	C	G	G	G
EPI_ISL_464200 20A	T	T	A	C	T	C	A	G	C	G	G	G
EPI_ISL_418390 20A	T	T	A	C	T	C	A	G	C	G	G	G
EPI_ISL_414523 20A	T	T	A	C	T	C	A	G	C	G	G	G
EPI_ISL_418397 20B	T	T	A	C	T	C	A	G	C	A	A	C
EPI_ISL_452220 20B	T	T	A	C	T	C	A	G	C	A	A	C
EPI_ISL_455326 20B	T	T	A	C	T	C	A	G	C	A	A	C
EPI_ISL_437095 20C	T	T	A	C	T	C	A	G	C	G	G	G
EPI_ISL_419602 20C	T	T	A	C	T	C	A	G	C	G	G	G
EPI_ISL_444856 20C DK	T	T	A	C	T	C	A	G	C	G	G	G
Mink_AD4_Farm1	T	T	G	C	T	T	T	G	T	A	A	C
Mink_AD6_Farm1	T	T	A	C	T	T	T	G	T	A	A	C
Mink_AD38_Farm 2	T	T	A	C	T	T	T	G	T	A	A	C
Mink_AD40_Farm 3	T	T	A	C	T	T	T	G	T	A	A	C
Mink_AL35_Farm3	T	T	A	C	T	T	T	G	T	A	A	C
H4	T	T	A	C	T	T	T	G	T	A	A	C
H5	T	T	A	C	T	T	T	G	T	A	A	C
H6	T	T	A	C	T	T	T	G	T	A	A	C
H7	T	T	A	T	T	T	T	G	T	A	A	C
H8	T	T	A	C	T	T	T	G	T	A	A	C
NC_045512 (Wuhan)	C	C	A	C	C	C	A	A	C	G	G	G

*Nucleotide differences from the Wuhan reference sequence are indicated in red. The highlighted changes indicate the location of the sequences from Denmark matching to clade 20B. Selected sequences containing specific mutations shared with the mink sequences from Denmark are also shown.

Appendix Table 2. Heterogeneity at indicated nt in SARS-CoV-2 from the Jutland region of Denmark

Nt	241	3037	5421	9534	14408	15656	22920	23403	25936	28881	28882	28883
Wuhan (NC045512.2)	C	C	A	C	C	C	A	A	C	G	G	G
Humans to 2020 Jul 1, n = 123	T	T	A	C	T	C>T	A>T	G	C>T	G>A	G>A	G>C
A	0	0	121	0	0	0	77	7	0	51	51	0
T	113	113	0	1	114	46	44	0	46	0	0	0
G	0	0	0	0	0	0	0	113	0	72	72	72
C	8	8	0	121	8	76	0	0	75	0	0	51
N	2	2	2	1	1	1	2	3	2	0	0	0
Humans to 2020 Jun 10, n = 63	T	T	A	C	T	C	A	G	C	G	G	G
A	0	0	62	0	0	0	61	3	0	4	4	0
T	60	59	0	0	60	0	0	0	0	0	0	0
G	0	0	0	0	0	0	0	58	0	58	58	58
C	3	3	0	62	3	63	0	0	63	0	0	4
N	0	1	1	1	0	0	2	2	0	1	1	1

*The predominant nt at the indicated positions in the genome of human samples (63) collected up to 10–06–20 or up to 01–07–20 (123 samples in total) is highlighted in yellow, high frequency variants are highlighted in green. The marked increase in variants in the human population at nt 15656, 22920, 25936 and at 28881–28883 between 10–06–20 and 01–07–20 is apparent. The nts present in the Wuhan reference sequence are indicated in black, the predominant variant at each position is indicated in red and highlighted in yellow, high frequency variants (>40%) are highlighted in green, and minority alternative nts at each position (if any) are indicated in blue.

.

Appendix Table 3. GISAID acknowledgment table

Virus name	Accession number	Collected
Romania/Bucuresti-4322/2020	EPI_ISL_468136	22–03–2020
Romania/Suceava-4682/2020	EPI_ISL_468137	22–03–2020
Poland/PL_P29/2020	EPI_ISL_455442	18–03–2020
Norway/1811/2020	EPI_ISL_420139	05–03–2020
CostaRica/02/2020	EPI_ISL_434535	16–03–2020
Thailand/61/2020	EPI_ISL_403962	08–01–2020
Jordan/SR-034/2020	EPI_ISL_429994	17–03–2020
Jordan/SR-045/2020	EPI_ISL_430003	16–03–2020
Jordan/SR-0335/2020	EPI_ISL_450187	08–04–2020
USA/CA2/2020	EPI_ISL_406036	22–01–2020
NewZealand/20VR2065/2020	EPI_ISL_456356	06–04–2020
NewZealand/20VR2085/2020	EPI_ISL_456369	12–04–2020
Austria/CeMM0013/2020	EPI_ISL_419666	11–03–2020
Austria/CeMM0017/2020	EPI_ISL_419670	16–03–2020
Austria/CeMM0020/2020	EPI_ISL_419673	22–03–2020
Austria/CeMM0146/2020	EPI_ISL_437994	28–02–2020
Austria/CeMM0147/2020	EPI_ISL_437995	28–02–2020
Austria/CeMM0156/2020	EPI_ISL_438002	03–03–2020
Austria/CeMM0167/2020	EPI_ISL_438012	06–03–2020
Austria/CeMM0180/2020	EPI_ISL_438022	09–03–2020
Austria/CeMM0186/2020	EPI_ISL_438028	11–03–2020
Austria/CeMM0188/2020	EPI_ISL_438029	13–03–2020
Austria/CeMM0189/2020	EPI_ISL_438030	13–03–2020
Austria/CeMM0191/2020	EPI_ISL_438032	13–03–2020
Austria/CeMM0235/2020	EPI_ISL_438066	19–03–2020
Austria/CeMM0265/2020	EPI_ISL_438094	23–03–2020
Austria/CeMM0267/2020	EPI_ISL_438096	24–03–2020
Austria/CeMM0279/2020	EPI_ISL_438106	25–03–2020
Germany/NW-HHU-03/2020	EPI_ISL_414498	26–02–2020
Germany/NW-HHU-07/2020	EPI_ISL_414506	27–02–2020
Germany/NW-HHU-32/2020	EPI_ISL_419549	15–03–2020
Germany/NW-HHU-36/2020	EPI_ISL_425120	16–03–2020
Germany/NW-HHU-49/2020	EPI_ISL_425133	10–03–2020
Germany/NW-HHU-52/2020	EPI_ISL_425136	14–03–2020
Taiwan/78/2020	EPI_ISL_428490	16–03–2020
Germany/MV-Cento-70004280/2020	EPI_ISL_459963	01–04–2020
Latvia/021/2020	EPI_ISL_450521	28–04–2020

Virus name	Accession number	Collected
Latvia/022/2020	EPI_ISL_450522	28–04–2020
France/N1620/2020	EPI_ISL_414624	26–02–2020
France/HF1465/2020	EPI_ISL_418218	21–02–2020
France/HF2150/2020	EPI_ISL_418224	08–03–2020
France/ARA12269/2020	EPI_ISL_419187	22–03–2020
France/Valence_532/2020	EPI_ISL_416749	04–03–2020
Portugal/PT0001b/2020	EPI_ISL_417986	03–03–2020
France/HF3295/2020	EPI_ISL_421511	23–03–2020
France/HF3677/2020	EPI_ISL_428359	23–03–2020
Germany/BY-ChVir-929/2020	EPI_ISL_406862	28–01–2020
Japan/P2–1/2020	EPI_ISL_419297	10–03–2020
Bangladesh/CHRF-0001/2020	EPI_ISL_437912	18–04–2020
Bangladesh/CHRF-0003/2020	EPI_ISL_468071	17–04–2020
France/B2335/2020	EPI_ISL_416504	02–03–2020
France/B5685/2020	EPI_ISL_443291	02–04–2020
France/B5434/2020	EPI_ISL_443279	01–04–2020
France/B5456/2020	EPI_ISL_443281	03–04–2020
France/BFC5012/2020	EPI_ISL_443262	02–04–2020
France/OCC-13/2020	EPI_ISL_434628	07–04–2020
France/OCC-14/2020	EPI_ISL_434629	07–04–2020
BosniaandHerzegovina/ChVir7343/2020	EPI_ISL_462451	19–03–2020
BosniaandHerzegovina/ChVir7346/2020	EPI_ISL_462453	20–03–2020
BosniaandHerzegovina/ChVir7347/2020	EPI_ISL_462454	20–03–2020
BosniaandHerzegovina/ChVir7349/2020	EPI_ISL_462456	20–03–2020
BosniaandHerzegovina/ChVir7365/2020	EPI_ISL_462467	26–03–2020
BosniaandHerzegovina/ChVir7370/2020	EPI_ISL_462471	28–03–2020
France/IDF2792/2020	EPI_ISL_420043	18–03–2020
Spain/CM-IBV-003903/2020	EPI_ISL_474831	19–05–2020
Kuwait/KU17/2020	EPI_ISL_416542	02–03–2020
Kuwait/KU09/2020	EPI_ISL_416541	02–03–2020
Iceland/170/2020	EPI_ISL_417548	17–03–2020
Belgium/ULG-10006/2020	EPI_ISL_421185	30–03–2020
Belgium/ULG-10024/2020	EPI_ISL_421198	30–03–2020
Belgium/ULG-10081/2020	EPI_ISL_424640	03–04–2020
Belgium/ULG-10088/2020	EPI_ISL_424646	04–04–2020
Belgium/ULG-10082/2020	EPI_ISL_424649	04–04–2020
Belgium/ULG-10170/2020	EPI_ISL_455958	11–05–2020
Belgium/ULG-10175/2020	EPI_ISL_455963	08–05–2020
Belgium/ULG-10182/2020	EPI_ISL_455970	10–05–2020
Belgium/ULG-10192/2020	EPI_ISL_471427	20–05–2020
Denmark/ALAB-HH11/2020	EPI_ISL_429270	10–03–2020
Denmark/ALAB-HH-104/2020	EPI_ISL_451990	08–03–2020
Denmark/ALAB-HH-156/2020	EPI_ISL_452031	19–03–2020
Denmark/ALAB-HH-177/2020	EPI_ISL_452045	14–04–2020
Denmark/ALAB-HH-213/2020	EPI_ISL_452068	27–04–2020
Denmark/ALAB-HH-233/2020	EPI_ISL_452077	28–04–2020
Denmark/ALAB-HH-244/2020	EPI_ISL_452082	19–04–2020
Denmark/ALAB-HH-262/2020	EPI_ISL_452093	14–04–2020
HongKong/HKPU52–3101/2020	EPI_ISL_419224	13–02–2020
France/IDF0372/2020	EPI_ISL_406596	23–01–2020
Italy/ABR-IZSGC-484/2020	EPI_ISL_457749	27–02–2020
Switzerland/ZH-UZH-1000477377/2020	EPI_ISL_413020	27–02–2020
Malaysia/188407/2020	EPI_ISL_417918	18–03–2020
Cyprus/006/2020	EPI_ISL_463746	11–04–2020
Cyprus/008/2020	EPI_ISL_463748	27–04–2020
England/CAMB-7FB83/2020	EPI_ISL_433484	17–04–2020
England/CAMB-7B853/2020	EPI_ISL_442170	05–04–2020
England/CAMB-8320F/2020	EPI_ISL_443398	14–04–2020
England/CAMB-1AD06E/2020	EPI_ISL_447960	30–04–2020
England/CAMB-1B3EF2/2020	EPI_ISL_473454	08–06–2020
England/CAMB-1B479C/2020	EPI_ISL_473483	03–06–2020
England/CAMB-1B493C/2020	EPI_ISL_473487	04–06–2020
Germany/BY-ChVir-1482/2020	EPI_ISL_450207	07–02–2020
Germany/BY-ChVir-1483/2020	EPI_ISL_450208	08–02–2020
Finland/13M33/2020	EPI_ISL_418390	13–03–2020
Finland/13M57/2020	EPI_ISL_418391	13–03–2020
Finland/13M65/2020	EPI_ISL_418395	13–03–2020
Finland/13M77/2020	EPI_ISL_418397	13–03–2020
Finland/13M82/2020	EPI_ISL_418399	13–03–2020

Virus name	Accession number	Collected
Finland/14M26/2020	EPI_ISL_418406	14–03–2020
Pakistan/NIH-45090/2020	EPI_ISL_468161	02–06–2020
Denmark/ALAB-SSI120/2020	EPI_ISL_429349	08–03–2020
Denmark/ALAB-SSI126/2020	EPI_ISL_429354	08–03–2020
Denmark/ALAB-SSI150/2020	EPI_ISL_429368	09–03–2020
Denmark/ALAB-SSI237/2020	EPI_ISL_429415	10–03–2020
Denmark/ALAB-SSI365/2020	EPI_ISL_429473	23–03–2020
Denmark/ALAB-SSI394/2020	EPI_ISL_429489	24–03–2020
Denmark/ALAB-SSI514/2020	EPI_ISL_429582	16–03–2020
Denmark/ALAB-SSI-1102/2020	EPI_ISL_437635	18–03–2020
Denmark/ALAB-SSI-133/2020	EPI_ISL_437642	08–03–2020
Denmark/ALAB-SSI-1406/2020	EPI_ISL_437651	04–04–2020
Denmark/ALAB-SSI-991/2020	EPI_ISL_437683	01–04–2020
Denmark/ALAB-SSI-1239/2020	EPI_ISL_444819	11–03–2020
Denmark/ALAB-SSI-1245/2020	EPI_ISL_444824	11–03–2020
Denmark/ALAB-SSI-1390/2020	EPI_ISL_444855	03–04–2020
Denmark/ALAB-SSI-1391/2020	EPI_ISL_444856	04–04–2020
Denmark/ALAB-SSI-735/2020	EPI_ISL_444881	27–03–2020
Denmark/ALAB-SSI-848/2020	EPI_ISL_444939	30–03–2020
Denmark/ALAB-SSI-856/2020	EPI_ISL_444944	29–03–2020
Denmark/ALAB-SSI-861/2020	EPI_ISL_444946	29–03–2020
Norway/2387/2020	EPI_ISL_449792	05–04–2020
Japan/OS-20–07–1/2020	EPI_ISL_410532	23–01–2020
Austria/Graz-MUG5/2020	EPI_ISL_437201	02–04–2020
Austria/Graz-MUG8/2020	EPI_ISL_437298	08–04–2020
Austria/Graz-MUG16/2020	EPI_ISL_437302	17–04–2020
SouthKorea/KCDC2002/2020	EPI_ISL_425118	30–01–2020
Netherlands/NA_32/2020	EPI_ISL_415489	13–03–2020
Netherlands/NoordBrabant_47/2020	EPI_ISL_415504	09–03–2020
Netherlands/Gelderland_10/2020	EPI_ISL_422638	20–03–2020
Netherlands/NA_137/2020	EPI_ISL_422681	19–03–2020
Netherlands/NA_194/2020	EPI_ISL_422729	23–03–2020
Netherlands/NA_195/2020	EPI_ISL_422730	24–03–2020
Netherlands/NoordBrabant_69/2020	EPI_ISL_422865	02–03–2020
Netherlands/ZuidHolland_76/2020	EPI_ISL_422944	26–03–2020
Netherlands/ZuidHolland_81/2020	EPI_ISL_422949	27–03–2020
Netherlands/ZuidHolland_83/2020	EPI_ISL_422951	27–03–2020
Netherlands/ZuidHolland_84/2020	EPI_ISL_422952	27–03–2020
Netherlands/NoordHolland_10001/2020	EPI_ISL_454750	27–02–2020
Netherlands/Utrecht_10026/2020	EPI_ISL_454775	01–04–2020
Netherlands/Utrecht_10030/2020	EPI_ISL_454779	04–04–2020
Netherlands/NA_592/2020	EPI_ISL_455289	09–04–2020
Netherlands/NoordBrabant_107/2020	EPI_ISL_455291	04–04–2020
Netherlands/Friesland_20/2020	EPI_ISL_460642	07–04–2020
Netherlands/Gelderland_22/2020	EPI_ISL_460658	16–04–2020
Netherlands/Gelderland_116/2020	EPI_ISL_460877	22–05–2020
Netherlands/Gelderland_143/2020	EPI_ISL_460903	22–05–2020
Netherlands/Gelderland_148/2020	EPI_ISL_460908	22–05–2020
Netherlands/Gelderland_151/2020	EPI_ISL_460911	22–05–2020
Netherlands/Gelderland_30/2020	EPI_ISL_460930	06–04–2020
Netherlands/Gelderland_62/2020	EPI_ISL_460961	18–05–2020
Netherlands/Gelderland_64/2020	EPI_ISL_460963	18–05–2020
Netherlands/Gelderland_67/2020	EPI_ISL_460966	18–05–2020
Netherlands/Gelderland_96/2020	EPI_ISL_460995	21–05–2020
Netherlands/Limburg_11/2020	EPI_ISL_460999	08–05–2020
Netherlands/NA_666/2020	EPI_ISL_461101	29–02–2020
Netherlands/NoordHolland_24/2020	EPI_ISL_461214	06–05–2020
Netherlands/Utrecht_32/2020	EPI_ISL_461230	23–04–2020
Netherlands/Zeeland_41/2020	EPI_ISL_461280	06–04–2020
Netherlands/Zeeland_6/2020	EPI_ISL_461288	02–04–2020
Latvia/03/2020	EPI_ISL_421655	25–03–2020
Latvia/07/2020	EPI_ISL_426287	30–03–2020
Latvia/08/2020	EPI_ISL_426288	30–03–2020
Brazil/AP-IEC-161167/2020	EPI_ISL_450873	17–03–2020
Chile/Santiago-05015/2020	EPI_ISL_468751	29–04–2020
Russia/SCPM-O-03/2020	EPI_ISL_451965	20–03–2020
BosniaandHerzegovina/04-Sarajevo/2020	EPI_ISL_467300	08–04–2020
Bangladesh/JUST-GC40.86/2020	EPI_ISL_475573	11–06–2020
India/GJ-GBRC174a/2020	EPI_ISL_467037	05–06–2020

Virus name	Accession number	Collected
Germany/HE-FFM4/2020	EPI_ISL_452220	02–03–2020
Sweden/20–08801/2020	EPI_ISL_475120	04–04–2020
Poland/1109201/2020	EPI_ISL_450525	28–03–2020
Poland/1109500/2020	EPI_ISL_450526	29–03–2020
Poland/1111628/2020	EPI_ISL_450530	01–04–2020
HongKong/VM20002849/2020	EPI_ISL_414571	22–02–2020
Belgium/ITM_C161/2020	EPI_ISL_450728	29–03–2020
Belgium/ITM_C237/2020	EPI_ISL_450732	01–04–2020
Chile/Santiago_74/2020	EPI_ISL_445370	05–04–2020
Spain/MD-IBV-003699/2020	EPI_ISL_467231	02–04–2020
Spain/MD-IBV-003702/2020	EPI_ISL_467235	02–04–2020
Chile/Chillan_2/2020	EPI_ISL_445331	12–03–2020
Norway/1989/2020	EPI_ISL_420147	10–03–2020
Brazil/SP-06/2020	EPI_ISL_414015	29–02–2020
Spain/CT-HUVH-VH9434/2020	EPI_ISL_444987	26–03–2020
Spain/PV-IBV-000792/2020	EPI_ISL_452722	09–03–2020
Spain/PV-IBV-000779/2020	EPI_ISL_452726	09–03–2020
Spain/PV-IBV-000760/2020	EPI_ISL_452731	10–03–2020
Spain/CN-ISCIII-201939/2020	EPI_ISL_455326	29–02–2020
Spain/AN-IBV-001895/2020	EPI_ISL_452453	15–03–2020
Spain/GA-IBV-002922/2020	EPI_ISL_474854	21–03–2020
Spain/AN-IBV-003033/2020	EPI_ISL_474935	06–04–2020
Spain/AN-ISCIII-201623/2020	EPI_ISL_455322	05–03–2020
Sweden/20–07833/2020	EPI_ISL_450819	24–04–2020
Sweden/20–14647/2020	EPI_ISL_469059	14–05–2020
Sweden/20–04631/2020	EPI_ISL_430847	27–02–2020
Iran/HGRC-2–2162/2020	EPI_ISL_437512	26–03–2020
India/WB-S52/2020	EPI_ISL_455672	03–05–2020
Russia/Moscow_PMVL-7/2020	EPI_ISL_470900	20–03–2020
Italy/LAZ-INMI1-cs/2020	EPI_ISL_410546	29–01–2020
Italy/LAZ-INMI4/2020	EPI_ISL_417922	28–02–2020
Senegal/003/2020	EPI_ISL_418206	28–02–2020
Senegal/016/2020	EPI_ISL_418207	02–03–2020
Croatia/I7-S21new/2020	EPI_ISL_454606	30–03–2020
Croatia/AU-S10new/2020	EPI_ISL_468656	09–04–2020
Poland/IHG_PAS_1_69/2020	EPI_ISL_450294	11–04–2020
Wuhan/IPBCAMS-WH-04/2019	EPI_ISL_403929	30–12–2019
Slovakia/SK-BMC5/2020	EPI_ISL_417879	06–03–2020
Slovakia/SK-BMC6/2020	EPI_ISL_417880	08–03–2020
Portugal/PT0049/2020	EPI_ISL_421452	18–03–2020
Brazil/DF-0001/2020	EPI_ISL_426580	13–03–2020
Israel/CVL-n-6051/2020	EPI_ISL_474961	09–04–2020
Italy/APU-UniMI-804/2020	EPI_ISL_469019	20–03–2020
Italy/APU-UniMI-809/2020	EPI_ISL_469020	19–03–2020
Italy/APU-UniMI-847/2020	EPI_ISL_469022	20–03–2020
Kuwait/KU005/2020	EPI_ISL_422426	16–03–2020
Jamaica/JM-CDC-0078/2020	EPI_ISL_450792	11–03–2020
Jamaica/JM-CDC-4376/2020	EPI_ISL_450794	11–03–2020
Pakistan/KHI1/2020	EPI_ISL_451958	16–03–2020
Japan/DP0134/2020	EPI_ISL_416577	15–02–2020
Japan/DP0482/2020	EPI_ISL_416606	16–02–2020
Sweden/20–50144/2020	EPI_ISL_454884	04–03–2020
Sweden/20–50155/2020	EPI_ISL_454888	04–03–2020
Sweden/20–50094/2020	EPI_ISL_455850	04–03–2020
Sweden/20–50259/2020	EPI_ISL_455897	07–03–2020
Sweden/20–50261/2020	EPI_ISL_469078	07–03–2020
Japan/Donner29/2020	EPI_ISL_469287	01–05–2020
Japan/Donner26/2020	EPI_ISL_438970	25–03–2020
Sweden/20–50130/2020	EPI_ISL_429141	06–03–2020
Sweden/RV-FOI-4/2020	EPI_ISL_428148	18–03–2020
Sweden/20–06909/2020	EPI_ISL_450811	06–04–2020
SouthKorea/KCDC03/2020	EPI_ISL_407193	25–01–2020
Belgium/BC-03016/2020	EPI_ISL_415157	01–03–2020
Belgium/MAC-0324142/2020	EPI_ISL_420399	24–03–2020
Belgium/FAE-030948/2020	EPI_ISL_420445	09–03–2020
Belgium/Rega-0329227/2020	EPI_ISL_458197	29–03–2020
Belgium/Rega-0329233/2020	EPI_ISL_458203	29–03–2020
Belgium/Rega-0330253/2020	EPI_ISL_458223	30–03–2020
Belgium/RJ-0507419/2020	EPI_ISL_462273	07–05–2020

Virus name	Accession number	Collected
Belgium/HI-0507421/2020	EPI_ISL_462275	07–05–2020
Belgium/rega-0407283/2020	EPI_ISL_464077	07–04–2020
Belgium/SA-0409296/2020	EPI_ISL_464090	09–04–2020
Sweden/20–51337/2020	EPI_ISL_475542	08–05–2020
Belgium/UGent-24/2020	EPI_ISL_425063	24–03–2020
Belgium/UGent-98/2020	EPI_ISL_468746	17–03–2020
Belgium/UGent-112/2020	EPI_ISL_475072	18–03–2020
France/IDF5986/2020	EPI_ISL_443305	13–04–2020
France/IDF5650/2020	EPI_ISL_443284	02–04–2020
France/IDF5655/2020	EPI_ISL_443285	02–04–2020
Canada/QC_AC6.1/2020	EPI_ISL_463904	05–04–2020
Canada/QC_BC5/2020	EPI_ISL_463968	09–04–2020
Switzerland/AG-SNRCI-29940361/2020	EPI_ISL_413999	27–02–2020
Switzerland/BL-SNRCI-29950902/2020	EPI_ISL_414021	27–02–2020
Luxembourg/LNS7991123/2020	EPI_ISL_421753	17–03–2020
Luxembourg/LNS6969569/2020	EPI_ISL_428943	01–04–2020
Luxembourg/LNS6854244/2020	EPI_ISL_428956	01–04–2020
Luxembourg/LNS9322962/2020	EPI_ISL_428961	03–04–2020
Luxembourg/LNS0756265/2020	EPI_ISL_429725	30–03–2020
Luxembourg/LNS3212178/2020	EPI_ISL_429728	26–03–2020
Luxembourg/LNS0481305/2020	EPI_ISL_429729	22–03–2020
Luxembourg/LNS7438855/2020	EPI_ISL_429730	09–04–2020
Luxembourg/LNS4107430/2020	EPI_ISL_429772	28–03–2020
Luxembourg/LNS7299024/2020	EPI_ISL_429775	23–03–2020
Luxembourg/LNS0994857/2020	EPI_ISL_429777	24–03–2020
Luxembourg/LNS9865959/2020	EPI_ISL_445056	22–04–2020
Luxembourg/LNS9063347/2020	EPI_ISL_445067	21–04–2020
Luxembourg/LNS7342327/2020	EPI_ISL_459905	12–05–2020
Luxembourg/LNS1874423/2020	EPI_ISL_419573	11–03–2020
Luxembourg/LNS3879580/2020	EPI_ISL_419585	14–03–2020
Luxembourg/LNS4836560/2020	EPI_ISL_419588	16–03–2020
Luxembourg/LNS4845603/2020	EPI_ISL_419589	15–03–2020
Luxembourg/LNS8188502/2020	EPI_ISL_419598	14–03–2020
Luxembourg/LNS9324837/2020	EPI_ISL_419602	12–03–2020
Luxembourg/LNS9627078/2020	EPI_ISL_419604	15–03–2020
Sweden/20–08719/2020	EPI_ISL_429135	02–04–2020
Sweden/20–08715/2020	EPI_ISL_450836	02–04–2020
Uruguay/UY-NYUMC869/2020	EPI_ISL_457965	03–04–2020
Peru/LIM-010/2020	EPI_ISL_415787	10–03–2020
Romania/283584/2020	EPI_ISL_455469	12–05–2020
Romania/284056/2020	EPI_ISL_455473	14–05–2020
Romania/284762/2020	EPI_ISL_455475	14–05–2020
Romania/284371/2020	EPI_ISL_455477	13–05–2020
Romania/Buzau-291946/2020	EPI_ISL_471416	01–06–2020
Romania/Buzau-293197/2020	EPI_ISL_471419	03–06–2020
Taiwan/CGMH-CGU-12/2020	EPI_ISL_417525	14–03–2020
Taiwan/CGMH-CGU-26/2020	EPI_ISL_452178	19–04–2020
Greece/12/2020	EPI_ISL_418264	18–03–2020
Greece/16/2020	EPI_ISL_418265	18–03–2020
Greece/38/2020	EPI_ISL_434460	23–03–2020
Greece/220_35357/2020	EPI_ISL_437887	18–03–2020
Greece/33_36910/2020	EPI_ISL_437893	29–03–2020
Greece/42_36236/2020	EPI_ISL_437899	23–03–2020
Greece/56_37161/2020	EPI_ISL_437911	31–03–2020
Poland/Pom4/2020	EPI_ISL_451645	02–05–2020
Italy/FVG-ICGEB-S5/2020	EPI_ISL_417419	01–03–2020
Italy/FVG-ICGEB-S18/2020	EPI_ISL_428854	30–03–2020
Italy/FVG-ICGEB-S1/2020	EPI_ISL_417418	01–03–2020
Italy/LAZ-INMI1-B2/2020	EPI_ISL_451299	04–02–2020
Italy/LAZ-INMI1-N/2020	EPI_ISL_451300	03–02–2020
Italy/LAZ-INMI11-B/2020	EPI_ISL_451304	23–03–2020
Kazakhstan/38716/2020	EPI_ISL_454575	09–05–2020
NewZealand/20VR1278/2020	EPI_ISL_456203	20–03–2020
Latvia/010/2020	EPI_ISL_437089	23–03–2020
Latvia/016/2020	EPI_ISL_437095	23–03–2020
England/LIVE-A0293/2020	EPI_ISL_472098	02–06–2020
England/LIVE-A0767/2020	EPI_ISL_472133	02–06–2020
Morocco/RMPS-04/2020	EPI_ISL_469052	30–03–2020
Australia/QLDID929/2020	EPI_ISL_420878	24–03–2020

Virus name	Accession number	Collected
Germany/BY-MVP-0028/2020	EPI_ISL_437227	13–04–2020
Germany/BY-MVP-0037/2020	EPI_ISL_437236	22–03–2020
Germany/BY-MVP-0040/2020	EPI_ISL_437239	02–04–2020
Germany/BY-MVP-0051/2020	EPI_ISL_437250	12–03–2020
Germany/BY-MVP-0055/2020	EPI_ISL_437254	17–03–2020
Germany/BY-MVP-0061/2020	EPI_ISL_437260	22–03–2020
Germany/BY-MVP-0070/2020	EPI_ISL_437266	26–03–2020
Germany/BY-MVP-0088/2020	EPI_ISL_437282	01–04–2020
Germany/BY-MVP-0098/2020	EPI_ISL_452104	04–04–2020
Germany/BY-MVP-0185/2020	EPI_ISL_466878	13–04–2020
Germany/BY-MVP-0218/2020	EPI_ISL_466891	22–04–2020
Germany/BY-MVP-0246/2020	EPI_ISL_466901	30–04–2020
Germany/BY-MVP-0247/2020	EPI_ISL_466902	30–04–2020
Germany/BY-MVP-0268/2020	EPI_ISL_466910	17–05–2020
Germany/BY-MVP-0277/2020	EPI_ISL_466920	25–05–2020
Germany/BY-MVP-0287/2020	EPI_ISL_466921	25–05–2020
Germany/BY-MVP-0289/2020	EPI_ISL_466922	25–05–2020
Germany/BY-MVP-0294/2020	EPI_ISL_466924	26–05–2020
Germany/BY-MVP-V2012622/2020	EPI_ISL_420911	19–03–2020
USA/MI-MDHHS-SC20321/2020	EPI_ISL_447118	27–03–2020
USA/MI-MDHHS-SC20413/2020	EPI_ISL_452287	14–03–2020
Turkey/HSGM-8001/2020	EPI_ISL_428720	21–03–2020
Turkey/HSGM-8964/2020	EPI_ISL_428723	22–03–2020
Turkey/HSGM-4505/2020	EPI_ISL_429867	17–03–2020
Turkey/HSGM-10241/2020	EPI_ISL_429871	23–03–2020
Turkey/HSGM-1428/2020	EPI_ISL_437311	27–03–2020
Turkey/HSGM-302/2020	EPI_ISL_437313	27–03–2020
Turkey/HSGM-510/2020	EPI_ISL_437314	26–03–2020
Turkey/HSGM-1027/2020	EPI_ISL_437317	27–03–2020
Turkey/HSGM-1490/2020	EPI_ISL_437319	19–03–2020
Turkey/HSGM-1492/2020	EPI_ISL_437320	19–03–2020
Turkey/HSGM-1476/2020	EPI_ISL_437324	19–03–2020
Turkey/HSGM-1458/2020	EPI_ISL_437327	19–03–2020
Turkey/HSGM-1014/2020	EPI_ISL_437331	25–03–2020
Turkey/HSGM-4698/2020	EPI_ISL_437332	18–03–2020
Turkey/HSGM-12059/2020	EPI_ISL_437335	25–03–2020
USA/MN-MDH-1071/2020	EPI_ISL_470751	03–06–2020
UnitedArabEmirates/L0184/2020	EPI_ISL_435121	25–02–2020
UnitedArabEmirates/L0904/2020	EPI_ISL_435126	25–02–2020
UnitedArabEmirates/L2409/2020	EPI_ISL_435131	25–02–2020
UnitedArabEmirates/L7356/2020	EPI_ISL_435140	12–03–2020
UnitedArabEmirates/L4711/2020	EPI_ISL_469278	24–03–2020
Russia/CRIE160583/2020	EPI_ISL_462149	30–03–2020
SouthAfrica/KRISP-0055/2020	EPI_ISL_467444	24–03–2020
SouthAfrica/KRISP-0131/2020	EPI_ISL_467483	13–05–2020
SouthAfrica/KRISP-0147/2020	EPI_ISL_467497	30–05–2020
Italy/LOM-INMI-13075-B/2020	EPI_ISL_451308	01–03–2020
Italy/LOM-INMI-BG-11639/2020	EPI_ISL_460080	28–02–2020
Italy/LOM-INMI-7070/2020	EPI_ISL_460086	24–02–2020
Italy/LOM-INMI-5925/2020	EPI_ISL_460091	22–02–2020
Italy/LOM-INMI-9675/2020	EPI_ISL_460095	26–02–2020
CzechRepublic/IAB_16/2020	EPI_ISL_426891	26–03–2020
CzechRepublic/IAB_20/2020	EPI_ISL_426894	27–03–2020
Thailand/Phuket_247/2020	EPI_ISL_447914	25–01–2020
Sweden/20–07295/2020	EPI_ISL_452235	15–04–2020
Sweden/20–06459/2020	EPI_ISL_445224	25–03–2020
Sweden/20–51678/2020	EPI_ISL_469069	25–05–2020
Sweden/20–07440/2020	EPI_ISL_434671	16–04–2020
Sweden/20–14696/2020	EPI_ISL_469060	15–05–2020
Kazakhstan/7263/2020	EPI_ISL_454571	25–03–2020
India/DL-NCDC-01711/2020	EPI_ISL_435066	18–03–2020
India/DL-NCDC-01757/2020	EPI_ISL_435068	18–03–2020
Singapore/3/2020	EPI_ISL_407988	01–02–2020
Vietnam/VR03–38142/2020	EPI_ISL_408668	24–01–2020
Bangladesh/BCSIR-NILMRC-103/2020	EPI_ISL_475084	07–06–2020
Malaysia/MKAK-CL-2020–5045/2020	EPI_ISL_416829	24–01–2020
Malaysia/MKAK-CL-2020–5096/2020	EPI_ISL_416885	30–01–2020
Singapore/9/2020	EPI_ISL_410715	04–02–2020
Singapore/10/2020	EPI_ISL_410716	04–02–2020

Virus name	Accession number	Collected
Singapore/97/2020	EPI_ISL_443189	18–03–2020
Singapore/352/2020	EPI_ISL_469107	28–05–2020
Brunei/2/2020	EPI_ISL_435674	11–03–2020
Brunei/4/2020	EPI_ISL_435676	21–03–2020
Brunei/1/2020	EPI_ISL_443187	11–03–2020
Guam/GU_NHG_03/2020	EPI_ISL_445000	20–03–2020
Nigeria/KW017-CV24/2020	EPI_ISL_455362	10–04–2020
Nigeria/OS075-CV12/2020	EPI_ISL_455423	29–03–2020
Nigeria/OS085-CV14/2020	EPI_ISL_455424	29–03–2020
Ghana/3177_S12/2020	EPI_ISL_422403	30–03–2020
CzechRepublic/ChVir1912/2020	EPI_ISL_416743	05–03–2020
USA/NY-NYUMC279/2020	EPI_ISL_428793	05–04–2020
Oman/205033013/2020	EPI_ISL_458116	01–05–2020
Oman/205028472/2020	EPI_ISL_458119	11–04–2020
Oman/RESP-20–837/2020	EPI_ISL_457704	24–02–2020
Sweden/20–07010/2020	EPI_ISL_434661	07–04–2020
Sweden/20–07480/2020	EPI_ISL_434673	16–04–2020
Norway/1380/2020	EPI_ISL_417484	26–02–2020
Norway/1493/2020	EPI_ISL_417488	29–02–2020
Italy/ABR-IZSGC-6193/2020	EPI_ISL_420568	23–03–2020
Italy/ABR-IZSGC-TE6222/2020	EPI_ISL_420583	23–03–2020
Italy/ABR-IZSGC-TE5056/2020	EPI_ISL_418257	17–03–2020
Vietnam/HCMC-35005/2020	EPI_ISL_450739	17–03–2020
SaudiArabia/KAUST-Madinah50/2020	EPI_ISL_437490	26–03–2020
SaudiArabia/KAUST-Makkah178/2020	EPI_ISL_437701	06–04–2020
Australia/QLD02/2020	EPI_ISL_407896	30–01–2020
Australia/NSW309/2020	EPI_ISL_451573	04–04–2020
Italy/ABR-IZSGC-TE4953/2020	EPI_ISL_418258	14–03–2020
Canada/ON-PHL-2273/2020	EPI_ISL_418383	2020–02
England/NORW-EC671/2020	EPI_ISL_457546	10–05–2020
Georgia/Tb-477/2020	EPI_ISL_415642	10–03–2020
Georgia/Tb-82/2020	EPI_ISL_415644	28–02–2020
Georgia/Tb-712/2020	EPI_ISL_416481	16–03–2020
Lebanon/S9_764/2020	EPI_ISL_450515	11–03–2020
NorthernIreland/NIRE-FB6F7/2020	EPI_ISL_448968	26–03–2020
Croatia/ZG-297–20/2020	EPI_ISL_451934	05–03–2020
England/200990724/2020	EPI_ISL_414006	28–02–2020
England/200990660/2020	EPI_ISL_414523	27–02–2020
England/20134018004/2020	EPI_ISL_423097	23–03–2020
England/20144009104/2020	EPI_ISL_423504	31–03–2020
England/20092000804/2020	EPI_ISL_464182	24–02–2020
England/20098017604/2020	EPI_ISL_464195	26–02–2020
England/20099000304/2020	EPI_ISL_464199	28–02–2020
England/20099000504/2020	EPI_ISL_464200	27–02–2020
England/201061453/2020	EPI_ISL_464411	27–02–2020
England/20149004004/2020	EPI_ISL_466098	02–04–2020
England/20242082604/2020	EPI_ISL_471520	06–06–2020
Indonesia/JK-EIJK-0317/2020	EPI_ISL_435282	19–03–2020
Indonesia/JK-EIJK-02/2020	EPI_ISL_437190	26–03–2020
Indonesia/JK-EIJK-07/2020	EPI_ISL_467376	24–04–2020
Sweden/20–51445/2020	EPI_ISL_455110	14–05–2020
Spain/AN-IBV-001214/2020	EPI_ISL_452558	24–03–2020
Spain/IB-IBV-002492/2020	EPI_ISL_467056	14–03–2020
Spain/IB-IBV-2571/2020	EPI_ISL_468980	07–04–2020
Spain/VC-FISABIO-54/2020	EPI_ISL_425178	28–02–2020
Spain/VC-FISABIO-521/2020	EPI_ISL_447472	26–03–2020
Spain/VC-FISABIO-147/2020	EPI_ISL_436242	18–03–2020
Spain/PV-IBV-2130/2020	EPI_ISL_468878	12–04–2020
Spain/AN-IBV-001927/2020	EPI_ISL_452447	11–03–2020
Italy/ABR-IZSGC-TE26539/2020	EPI_ISL_436727	27–04–2020
Italy/ABR-IZSGC-TE13858/2020	EPI_ISL_435152	09–04–2020
Singapore/5/2020	EPI_ISL_410536	06–02–2020
Sweden/20–08047/2020	EPI_ISL_454495	08–03–2020
Sweden/20–08498/2020	EPI_ISL_475105	25–03–2020
Norway/Trondheim-S4/2020	EPI_ISL_450346	25–03–2020
Norway/Trondheim-S12/2020	EPI_ISL_450349	29–03–2020
Hangzhou/ZJU-02/2020	EPI_ISL_416042	26–01–2020
Russia/Yakutia-73709/2020	EPI_ISL_428874	20–03–2020
Russia/Moscow-67609/2020	EPI_ISL_428882	16–03–2020

Virus name	Accession number	Collected
Crimea/SRC-80603/2020	EPI_ISL_428901	23–03–2020
Russia/Buryatia-87105/2020	EPI_ISL_428920	30–03–2020
Norway/2065/2020	EPI_ISL_420149	09–03–2020
CzechRepublic/NRL_2312/2020	EPI_ISL_471544	14–03–2020
CzechRepublic/NRL_2554/2020	EPI_ISL_471547	11–03–2020
Iceland/297/2020	EPI_ISL_417626	18–03–2020
Iceland/303/2020	EPI_ISL_417632	18–03–2020
Iceland/325/2020	EPI_ISL_417654	18–03–2020
Iceland/157/2020	EPI_ISL_417804	13–03–2020
Iceland/167/2020	EPI_ISL_417809	15–03–2020
Iceland/222/2020	EPI_ISL_417837	16–03–2020
Iceland/53/2020	EPI_ISL_417851	05–03–2020
Iceland/56/2020	EPI_ISL_417852	06–03–2020
Iceland/91/2020	EPI_ISL_417873	10–03–2020
Iceland/436/2020	EPI_ISL_424460	20–03–2020
Iceland/470/2020	EPI_ISL_424493	21–03–2020
Taiwan/TSGH-23/2020	EPI_ISL_436108	02–04–2020
Uganda/UG001/2020	EPI_ISL_451183	25–03–2020
Uganda/UG017/2020	EPI_ISL_451199	20–04–2020
Italy/VEN-IZSVe-23–50/2020	EPI_ISL_452185	26–03–2020
Italy/VEN-IZSVe-31–19/2020	EPI_ISL_452188	31–03–2020
Sweden/20–51061/2020	EPI_ISL_475122	07–04–2020
Canada/ON-UHTC_0051/2020	EPI_ISL_464063	03–04–2020
Austria/CeMM0058/2020	EPI_ISL_437938	03–04–2020
Austria/CeMM0101/2020	EPI_ISL_437962	20–03–2020
Austria/CeMM0113/2020	EPI_ISL_437971	18–03–2020
Switzerland/BL-UHB-42169310/2020	EPI_ISL_418275	27–02–2020
Croatia/1560_Split/2020	EPI_ISL_454583	18–03–2020
Norway/1526/2020	EPI_ISL_417485	02–03–2020
Norway/2093/2020	EPI_ISL_420310	16–03–2020
Switzerland/GE-HUG-VD0503/2020	EPI_ISL_415459	29–02–2020
Switzerland/GE-HUG-6065/2020	EPI_ISL_429220	06–04–2020
HongKong/HKU-903b/2020	EPI_ISL_434566	27–01–2020
Greece/222_33921/2020	EPI_ISL_447643	22–03–2020
Greece/227_35969/2020	EPI_ISL_447644	11–03–2020
Greece/34_36284/2020	EPI_ISL_447832	24–03–2020
HongKong/VM2003179/2020	EPI_ISL_450405	08–03–2020
Portugal/PT0144/2020	EPI_ISL_453860	27–03–2020
Portugal/PT0159/2020	EPI_ISL_453875	27–03–2020
Portugal/PT0160/2020	EPI_ISL_453876	27–03–2020
Portugal/PT0199/2020	EPI_ISL_453915	28–03–2020
Portugal/PT0247/2020	EPI_ISL_453961	02–04–2020
Portugal/PT0268/2020	EPI_ISL_453982	04–04–2020
Portugal/PT0275/2020	EPI_ISL_453989	05–04–2020
Portugal/PT0321/2020	EPI_ISL_454037	15–03–2020
Portugal/PT0395a/2020	EPI_ISL_454114	18–04–2020
Portugal/PT0460/2020	EPI_ISL_454184	17–04–2020
Portugal/PT0466/2020	EPI_ISL_454190	18–04–2020
Portugal/PT0480/2020	EPI_ISL_454204	27–03–2020
Portugal/PT0487/2020	EPI_ISL_454211	01–04–2020
Portugal/PT0496/2020	EPI_ISL_454220	30–04–2020
Portugal/PT0509/2020	EPI_ISL_454233	19–03–2020
Portugal/PT0538/2020	EPI_ISL_454262	07–04–2020
Portugal/PT0539/2020	EPI_ISL_454263	07–04–2020
Portugal/PT0548/2020	EPI_ISL_454272	02–05–2020
Portugal/PT0580/2020	EPI_ISL_454303	25–03–2020
Portugal/PT0616/2020	EPI_ISL_454339	17–03–2020
Portugal/PT0549/2020	EPI_ISL_455627	03–04–2020
Zhejiang/OS5/2020	EPI_ISL_455689	24–03–2020
Turkey/IMU-SP-02/2020	EPI_ISL_460617	16–04–2020
Turkey/IMU-SP-01/2020	EPI_ISL_460618	15–04–2020
Sweden/20–50591/2020	EPI_ISL_475118	01–04–2020
Sweden/20–51516/2020	EPI_ISL_475526	17–05–2020
Turkey/ERAGEM-001/2020	EPI_ISL_424366	17–03–2020
Serbia/NP363–04/2020	EPI_ISL_437436	04–04–2020
Australia/VIC81/2020	EPI_ISL_419793	14–03–2020
Australia/VIC876/2020	EPI_ISL_427131	05–04–2020
Italy/ABR-IZSGC-TE12759/2020	EPI_ISL_435147	08–04–2020
Switzerland/ZH-ETHZ-100093/2020	EPI_ISL_451685	13–03–2020

Virus name	Accession number	Collected
Switzerland/GR-ETHZ-100147/2020	EPI_ISL_451731	16–03–2020
Switzerland/VD-ETHZ-100805/2020	EPI_ISL_451755	25–03–2020
Switzerland/BS-ETHZ-101147/2020	EPI_ISL_451814	31–03–2020
Switzerland/ZH-ETHZ-101172/2020	EPI_ISL_451835	31–03–2020
Switzerland/ZH-ETHZ-110439/2020	EPI_ISL_451864	07–04–2020
Switzerland/BL-ETHZ-110482/2020	EPI_ISL_451894	07–04–2020
Switzerland/VD-ETHZ-110507/2020	EPI_ISL_451913	07–04–2020
Switzerland/TI-ETHZ-100043/2020	EPI_ISL_466966	11–03–2020
Switzerland/TI-ETHZ-100049/2020	EPI_ISL_466971	11–03–2020
Switzerland/SZ-ETHZ-100062/2020	EPI_ISL_466984	12–03–2020
Switzerland/ZH-ETHZ-120244/2020	EPI_ISL_468303	04–05–2020
DRC/KN-0054/2020	EPI_ISL_417437	17–03–2020
DRC/214/2020	EPI_ISL_420030	21–03–2020
DRC/1324/2020	EPI_ISL_435033	07–04–2020
DRC/1151/2020	EPI_ISL_447596	05–04–2020
Poland/PL_P10/2020	EPI_ISL_451971	01–04–2020
Poland/PL_P14/2020	EPI_ISL_451975	31–03–2020
Poland/PL_P18/2020	EPI_ISL_451979	31–03–2020
Poland/PL_P24/2020	EPI_ISL_451985	29–03–2020
Poland/PL_P2/2020	EPI_ISL_428925	28–03–2020
Poland/PL_P7/2020	EPI_ISL_428930	29–03–2020
Hungary/SRC-00066/2020	EPI_ISL_435403	20–03–2020
Hungary/SRC-00067/2020	EPI_ISL_435404	20–03–2020
Hungary/SRC-02801w/2020	EPI_ISL_435428	25–03–2020
Hungary/mbl1/2020	EPI_ISL_416426	17–03–2020
Bangladesh/icddrb-2083/2020	EPI_ISL_470801	07–06–2020
Scotland/EDB5590/2020	EPI_ISL_461759	25–05–2020
Scotland/EDB6244/2020	EPI_ISL_473926	21–05–2020
Scotland/EDB6322/2020	EPI_ISL_473935	02–06–2020
England/SHEF-C09D4/2020	EPI_ISL_475343	28–04–2020
England/SHEF-C0D50/2020	EPI_ISL_475348	30–05–2020
Cambodia/0012/2020	EPI_ISL_411902	27–01–2020
Wales/PHWC-162AB2/2020	EPI_ISL_473158	20–05–2020
Wales/PHWC-163161/2020	EPI_ISL_473254	27–05–2020
Russia/StPetersburg-RII4546V/2020	EPI_ISL_427314	02–04–2020
Russia/StPetersburg-RII4584S/2020	EPI_ISL_427316	03–04–2020
Russia/StPetersburg-RII4332S/2020	EPI_ISL_427339	25–03–2020
Russia/StPetersburg-RII4938S/2020	EPI_ISL_430073	08–04–2020
Russia/StPetersburg-RII5047S/2020	EPI_ISL_430087	10–04–2020
Russia/StPetersburg-RII5642S/2020	EPI_ISL_430095	14–04–2020
Russia/StPetersburg-RII7464S/2020	EPI_ISL_450252	20–04–2020
Russia/StPetersburg-RII7603S/2020	EPI_ISL_450263	21–04–2020
Russia/StPetersburg-RII8393S/2020	EPI_ISL_450279	22–04–2020
Russia/StPetersburg-RII8402S/2020	EPI_ISL_450281	22–04–2020
Russia/StPetersburg-RII8913S/2020	EPI_ISL_450290	23–04–2020
USA/WI1/2020	EPI_ISL_408670	31–01–2020
Sweden/20–07666/2020	EPI_ISL_445238	20–04–2020

*We gratefully acknowledge the authors from the originating laboratories responsible for obtaining the specimens and the submitting laboratories where genetic sequence data were generated and shared via the GISAID Initiative, on which this research is based.

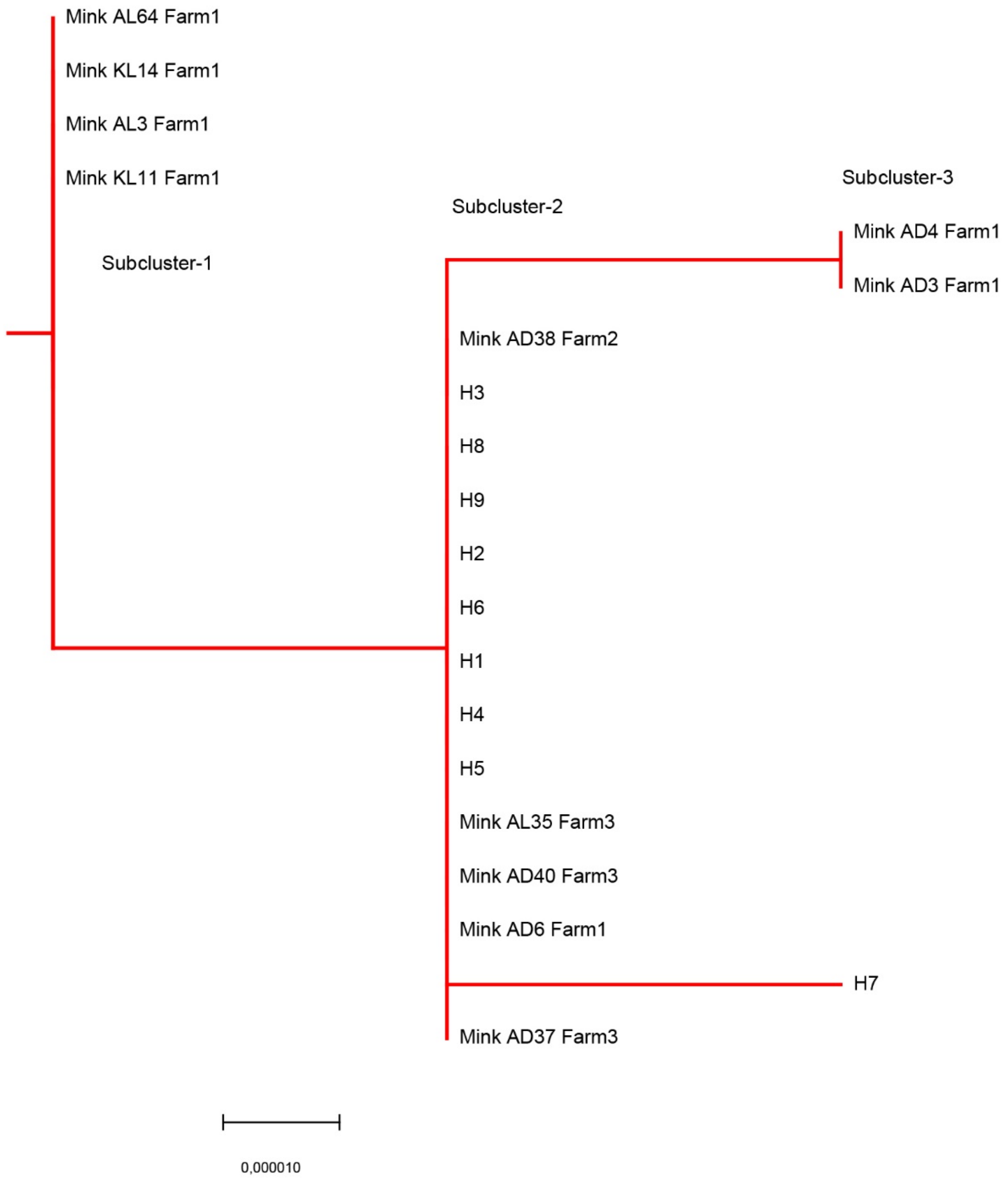

Appendix Figure. Phylogenetic tree showing the relationships between SARS-CoV-2 full genome sequences in farms 1, 2, and 3 and connected local human cases (H1–H9). Three subclusters are indicated, which differ by sequence changes at nt positions 5421 and 22920. Scale bar indicates nucleotide substitutions per site.
